# A possible genetic association between obesity and colon cancer in females

**DOI:** 10.3389/fendo.2023.1189570

**Published:** 2023-08-30

**Authors:** Xiao-li Zhang, Xin-feng Zhang, Yuan Fang, Meng-li Li, Ruo Shu, Yi Gong, Hua-you Luo, Yan Tian

**Affiliations:** ^1^ Department of Gastrointestinal and Hernia Surgery, The First Affiliated Hospital of Kunming Medical University, Kunming, China; ^2^ Department of Hepatobiliary Transplantation, The First Affiliated Hospital of Kunming Medical University, Kunming, China; ^3^ Department of Respiratory Medicine, Affiliated Hospital of Yunnan University, Kunming, China; ^4^ Basic Medical Sciences, Kunming Medical University, Kunming, China

**Keywords:** obesity, colon cancer, women, GEO, genes

## Abstract

**Object:**

There is mounting clinical evidence that an increase in obesity is linked to an increase in cancer incidence and mortality. Although studies have shown a link between obesity and colon cancer, the particular mechanism of the interaction between obesity and colon cancer in females remains unknown. The goal of this work is to use bioinformatics to elucidate the genetic link between obesity and colon cancer in females and to investigate probable molecular mechanisms.

**Methods:**

GSE44076 and GSE199063 microarray datasets were obtained from the Gene Expression Omnibus (GEO) database. In the two microarray datasets and healthy controls, the online tool GEO2R was utilized to investigate the differential genes between obesity and colon cancer. The differential genes (DEGs) identified in the two investigations were combined. Gene Ontology (GO) and Kyoto Encyclopedia of Genes and Genomes (KEGG) pathway enrichment studies were performed on the DEGs. The STRING database and Cytoscape software were then used to build protein-protein interaction (PPI) networks to discover hub genes. NetworkAnalyst was also used to build networks of target microRNAs (miRNAs) and hub genes, as well as networks of transcriptions.

**Results:**

Between the two datasets, 146 DEGs were shared. The DEGs are primarily enriched in inflammatory and immune-related pathways, according to GO analysis and KEGG. 14 hub genes were identified via PPI building using the Cytoscape software’s MCODE and CytoNCA plug-ins: TYROBP, CD44, BGN, FCGR3A, CD53, CXCR4, FN1, SPP1, IGF1, CCND1, MMP9, IL2RG, IL6 and CTGF. Key transcription factors for these hub genes include WRNIP1, ATF1, CBFB, and NR2F6. Key miRNAs for these hub genes include hsa-mir-1-3p, hsa-mir-26b-5p, hsa-mir-164a-5p and hsa-mir-9-5p.

**Conclusion:**

Our research provides evidence that changed genes are shared by female patients with colon cancer and obesity. Through pathways connected to inflammation and the immune system, these genes play significant roles in the emergence of both diseases. We created a network between hub genes and miRNAs that target transcription factors, which may offer suggestions for future research in this area.

## Introduction

In developed nations, a considerable fraction of the population is overweight or obese. Body mass index (BMI), which employs ranges of 18.5-24.9 for normal, 25-29.9 for overweight, and 30 for obesity, is typically used to measure obesity (1). Obesity prevalence has significantly increased during the previous ten to fifteen years and has already reached epidemic levels, affecting 2 billion individuals (2, 3). Due to an increase in caloric intake and a decrease in physical activity, there is a persistent rise in the incidence of related cancers. This is thought to be the cause of the ongoing rise in the obese and overweight population (4). Obesity is more prevalent in women than in males (5), and up to 20% of cancer deaths in women are thought to be attributable to it (6).

In developed countries, colon cancer (CC) is the second most prevalent malignant disease (7). Colonic epithelial cells, which are positioned in the organ’s lumen and self-renew every five days from cryptic stem cells at the base of the colonic epithelial cell crypt, are the source of CC (8). There may be a variety of variables that affect cellular self-renewal and lead to the formation of CC. These variables include one’s way of life, food and genetic alterations (9), which account for 15–30% of cases of CC and are brought on by oncogene overexpression and tumor suppressor gene inactivation (10). There is still a sizable number of CCs that are irregular and linked to environmental or lifestyle variables. Patients lack a definite genetic susceptibility (11, 12). CC is the most prevalent type of nutrition-related cancer in the general population, according to research (13). Hypertrophic expansion of fat adipose tissue brought on by overeating shares many characteristics with solid tumor growth. Obese people’s adipose tissue contains adipocyte precursor cells that accelerate tumor growth by producing more endothelial precursor cells, pericytes and adipocytes, which results in angiogenesis and the growth of cancer cells in living organisms (14).

Obese individuals have hyperinsulinemia, insulin resistance, hormonal dysregulation, a chronic inflammatory state and a dysregulated energy metabolism. These modifications put obese people at higher risk for cancer and a poor prognosis (15, 16). Adipose tissue is abundant in the tumor mesenchyme and adipocytes operate as endocrine cells that secrete signaling molecules such adipokines, pro-inflammatory cytokines and pro-angiogenic proteins to aid in the development and growth of tumors (17). For instance, through activation of the prostaglandin and insulin-like growth factor pathways, long-term persistent chronic inflammation and insulin resistance increase colon cell proliferation and block programmed cell death or apoptosis. The Wnt pathway is particularly rich in proteins involved in the control of cell polarity, mitotic activity and fertility, which is closely associated with colon carcinogenesis (18). Additionally, visceral adipocytes released greater amounts of lactate dehydrogenase (19), interleukin (IL)-6, IL-8 and tumor necrosis factor alpha (TNF). These inflammatory substances activate signaling pathways associated with NF-κB, STAT3, phosphatidylinositol 3-kinase/threonine kinase Akt (PI3K/Akt), mechanistic target of rapamycin/mitogen-activated protein kinase (mTOR/MAPK/p38) and other pro-inflammatory pathways. The development of metastases and associated cancer cell proliferation, invasion, angiogenesis and cell survival are all mediated by the activation of these pathways (4, 20–22). In contrast, while most clinical investigations have identified a significant link between weight and CC risk in males, there has been little to no evidence of this relationship in women (23–27). Therefore, it is crucial to research the relationship between female obesity and CC. Understanding the genetic relationships between CC and obesity may offer crucial mechanistic insights into CC in the setting of female obesity.

With the advancement of information technology in biology, increased gene screening in a genomic context has been made possible in recent years. To determine the differences between CC and obesity, this work employed two original microarray datasets retrieved from Gene Expression Omnibus (GEO, http://www.ncbi.nlm.nih.gov/geo). The detected DEGs were then examined using gene ontology (GO) analysis, KEGG pathway analysis and protein-protein interaction (PPI) analysis. Then, using the Cytoscape program, we predicted the probable transcription factors and miRNAs of hub genes and created a network that displayed the interactions between transcription factors, miRNAs and genes.

## Materials and methods

### Data download

The two datasets mentioned above were downloaded from the GEO website (https://www.ncbi.nlm.nih.gov/geo/). For the GSE44076 dataset, we chose 27 female CC patients and 23 healthy female controls; for the GSE199063 dataset, we chose 50 female obese patients and 28 healthy female controls. **Table 1** displays the dataset’s specifics. Without further authorization, the data were downloaded from the official platform.

**Table 1:** Details of the two dataset clusters.

Diseases	Serial number	Platform	Tissue Source	Patient group	Control group	Total number
Colon cancer	GSE44076	GPL13667	tumor samples healthy colon mucosae	27	23	50
obesity	GSE199063	GPL23126	Adipose tissue	50	28	78

GSE44076: normal adjacent mucosa and tumor samples from 98 individuals and 50 healthy colon mucosae. However, we only selected female patients who met the criteria for our study. GSE199063: adipose tissue from a cohort of obese women. Adipose tissue biopsies were obtained from the control group(n = 28),before RYGB(n = 50), and then 2(n = 49) and 5 years (n = 38)thereafter.

### Screening of differential genes between the two datasets

Using the online biometric analysis tool GEO2R (https://www.ncbi.nlm.nih.gov/geo/geo2r/), which is available on the official GEO website, differentially expressed genes in the two datasets were analyzed. Volcano plots were downloaded to further screen the DEGs between the two datasets with a screening threshold of|log2 FC | ≥ 1, *P* < 0.05. For overlapping DEGs between female obesity and female CC, the resulting differential genes between the two datasets were visualized using Venn plots from the Venn online platform (http://bioinformatics.psb.ugent.be/webtools/Venn/). The analysis of the obtained overlapped DEGs then continued.

### Functional enrichment analysis of DEGs

With the use of the R program, the aforementioned overlapping DEGs were analyzed for GO enrichment, including biological process (BP), cellular component (CC) and molecular function (MF) with an adjusted *P* value < 0.05.

### Pathway enrichment analysis of DEGs

The KEGG pathway enrichment analysis of the aforementioned overlapping DEGs was performed using the R package with an adjusted *P* value < 0.05.

### Protein interaction network analysis of DEGs

To further examine the relationships between DEGs, the aforementioned list of overlapping DEGs was imported into the STRING (http://string-db.org) database. To download the list of protein mediators and subsequently use Cytoscape software to display and analyze the PPI network, an interaction score of at least 0.4 was deemed significant. Key protein expression molecules were screened using the Cytoscape plugin Minimal Common Oncology Data Elements (MCODE), and PPI network was obtained. The PPI network was screened for hub genes using the number-centered method with the CytoNCA plug-in. The 14 genes that overlap between the two calculations made by the plug-ins were then subjected to the next step of study.

### MiRNAs and transcription factors associated with hub genes

The miRNAs, transcription factors, and miRNAs-transcription factors-gene connections of hub genes were created using the NetworkAnalyst program (version 3.0, available at https://www.networkanalyst.ca/). Cytoscape software was used to view and evaluate the data.

## Results

### Identification of DEGs in obesity and colon cancer

**Figure 1:**
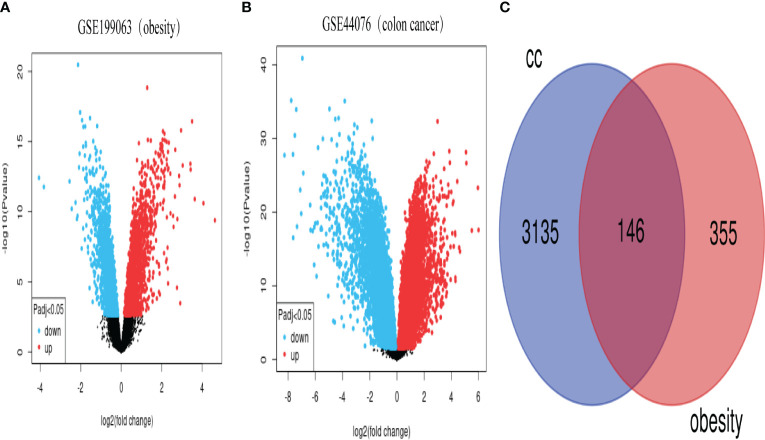
Volcano and Venn plots of differentially expressed genes. **(A)**, genes differentially expressed between obese and control in the GSE199063 dataset (red dots represent up-regulated genes, blue dots represent down-regulated genes). **(B)**, genes differentially expressed between colon cancer and healthy control in the GSE44076 dataset (red dots represent up-regulated genes, blue dots represent down-regulated genes). **(C)**, Number of genes overlapping differentially expressed genes in the above two datasets. (CC, colon cancer).

From the NCBI GEO database (https://www.ncbi.nlm.nih.gov/geo/), we retrieved the GSE199063 dataset on obesity and the GSE44076 dataset on CC. 355 DEGs were found in the GSE199063 dataset and 3135 DEGs were found in the GSE44076 dataset after modifying the threshold screening for *P* value < 0.05 and |log2 FC | >1.0. The GEO 2R web tool Volcano Plot was used to visualize the data results. The overlapped 146 genes were then obtained by intersecting the two datasets. The data outcomes were shown graphically in **Figure 1**.

### GO analysis of overlapping DEGs

**Figure 2:**
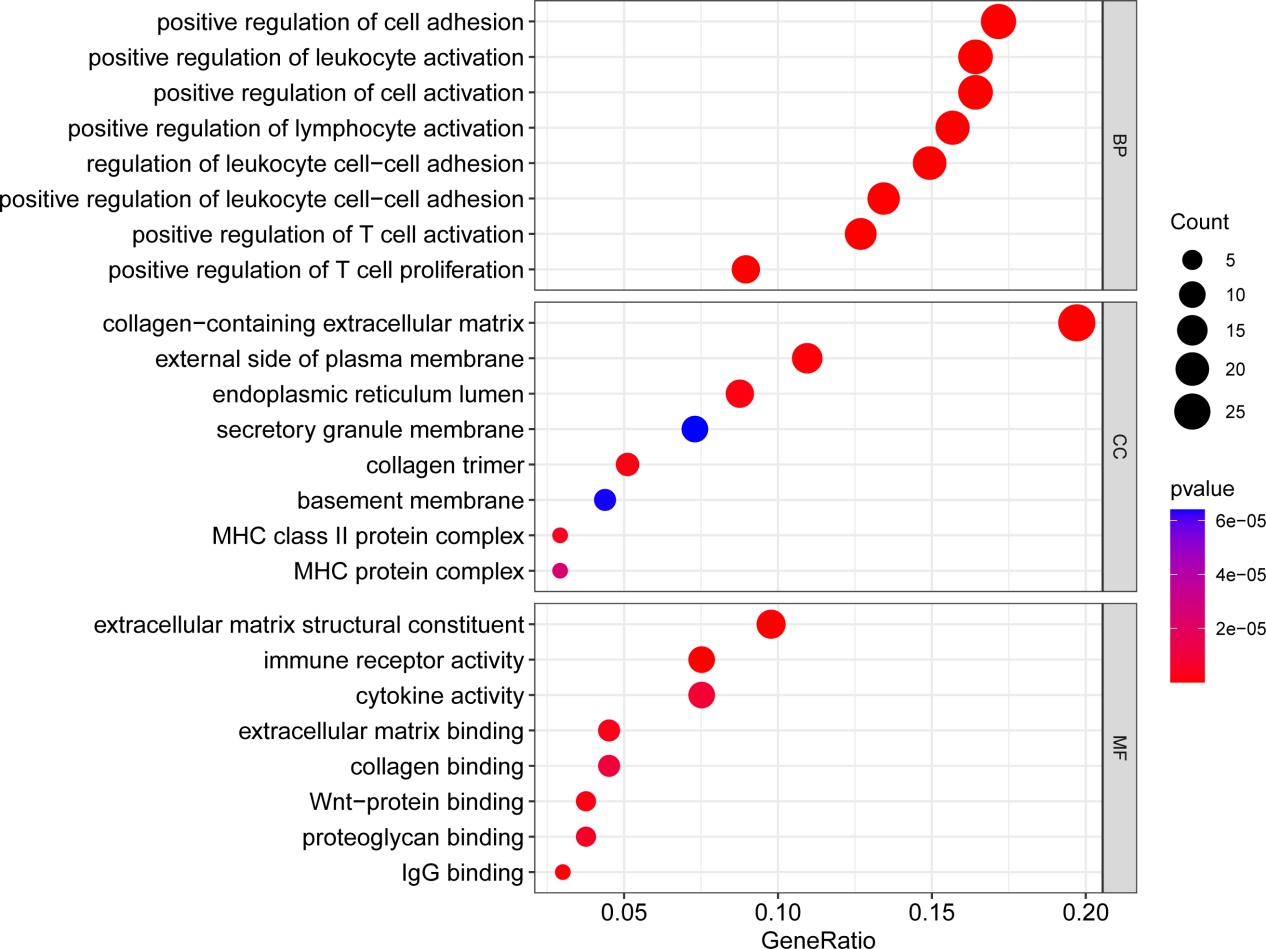
Bubble plot of GO enrichment analysis results. x-axis indicates the proportion of genes per functional term. y-axis indicates the annotated terms of gene enrichment. The circle size represents the number of genes: the larger the circle, the higher the number of genes. The circle color represents the adjusted P value: the redder the color, the higher the degree of gene enrichment. MF, Molecular Function; BP, Biological Process; CC, Cellular Component.

We used the cluster Profiler tool in R to analyze the GO enrichment function of overlapping DEGs in order to understand the function of these genes. The molecular function (MF), biological process (BP) and cellular component (CC) make up the three parts of the GO enrichment function. After the *P* < 0.05 screening adjustment, the following results were obtained: BP functions mainly include positive regulation of cell adhesion, positive regulation of leukocyte activation, positive regulation of cell activation, positive regulation of lymphocyte activation, regulation of leukocyte cell-cell adhesion, positive regulation of leukocyte cell-cell adhesion, positive regulation of T cell activation, positive regulation of T cell adhesion. CC functions mainly include collagen-containing extracellular matrix, external side of plasma membrane, endoplasmic reticulum lumen, secretory granule membrane, collagen trimer, basement membrane, MHC class II protein complex, MHC protein complex. MF functions mainly include extracellular matrix structural constituent, immune receptor activity, cytokine activity, extracellular matrix binding, collagen binding, wnt-protein binding, proteoglycan binding and IgG binging. (**Figure 2**; **Supplementary Figure 2S1**, **Supplementary Figure 2S2**, **Supplementary Table S1**)

### KEGG analysis of overlapping DEGs

**Figure 3:**
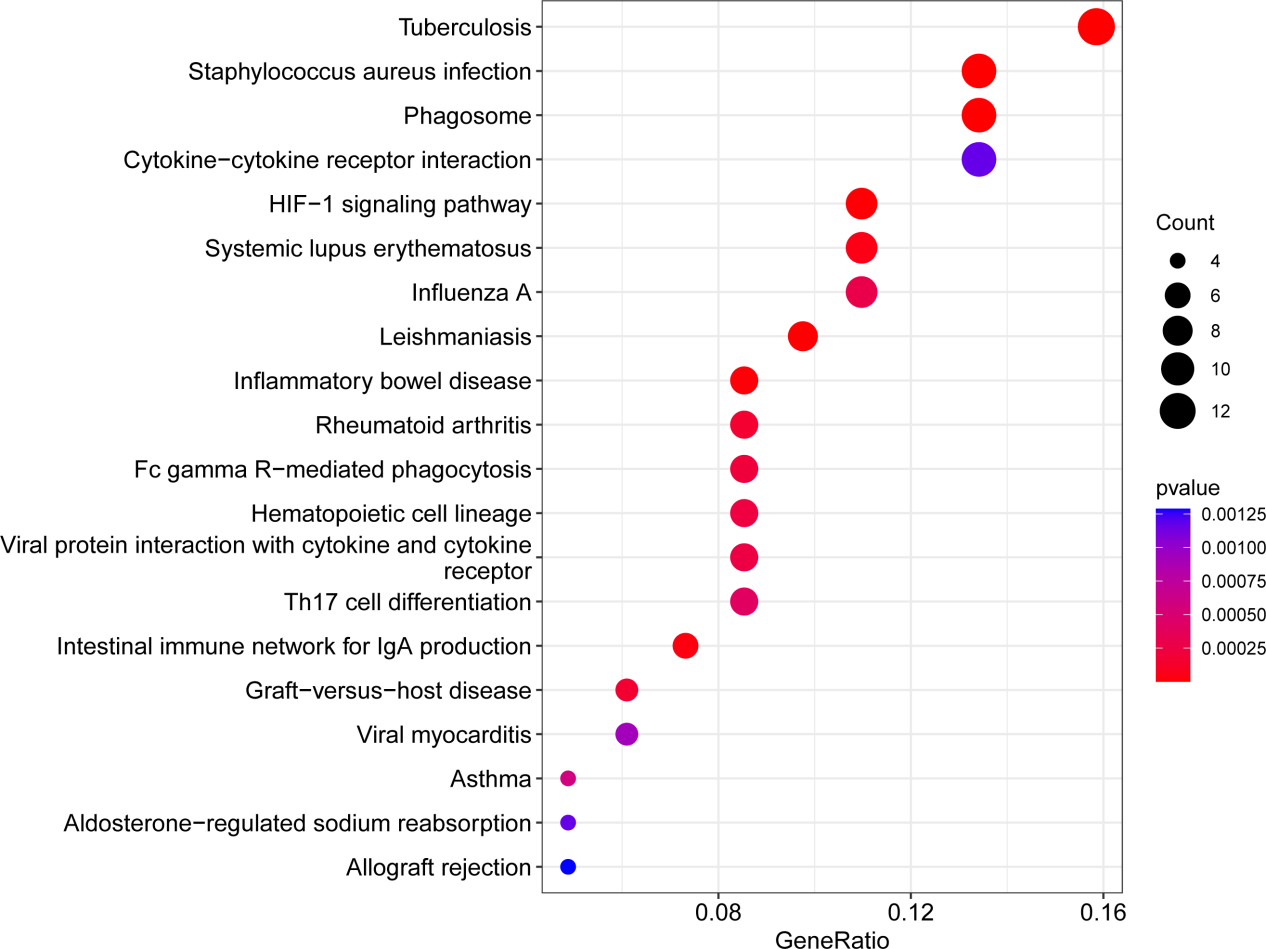
Bubble plot of KEGG enrichment analysis results. x-axis indicates the proportion of genes per functional term. y-axis indicates the annotated terms of gene enrichment. The circle size represents the number of genes: the larger the circle, the higher the number of genes. The circle color represents the adjusted P value: the redder the color, the higher the degree of gene enrichment.

**Figure 4:**
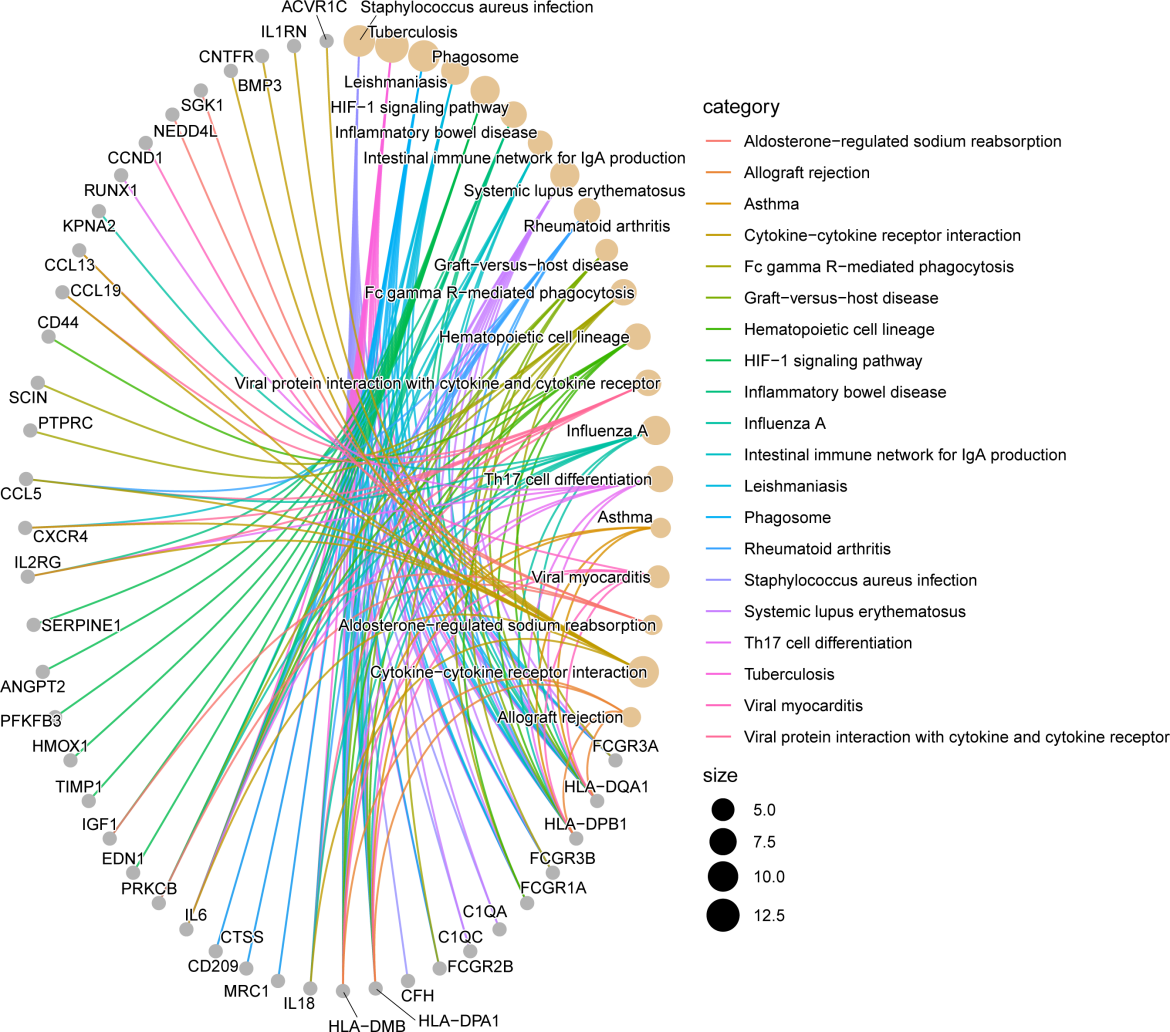
Network diagram of KEGG enrichment analysis results. The line segments connect genes and enrichment pathways, and different colors represent different enrichment pathways. The size of the circles represents the different number of connected line segments, the larger the circle, the more genes and pathways are connected, the gray circle represents genes, the yellow circle represents pathways.

We evaluated the KEGG enrichment function of these genes using the cluster Profiler program in R software in order to comprehend the enrichment pathway of overlapping DEGs. The following outcomes were attained following adjustment of the *P* < 0.05 screen: Tuberculosis, Staphylococcus aureus infection, Phagosome, Cytokine-cytokine receptor interaction, HIF-1 signaling pathway, Systemic lupus erythematosus, Influenza A, Leishmaniasis, Inflammatory bowel disease, Rheumatoid arthritis, Fc gamma R-mediated phagocytosis, Hematopoietic cell lineage, Viral protein interaction with cytokine and cytokine receptor, Th17 cell differentiation, Intestinal immune network for IgA production, Graft-versus-host disease, Viral myocarditis, Asthma, Aldosterone-regulated sodium reabsorption, Allograft rejection. (**Figures 3**, **4**; **Supplementary Figure 3S1**, **Supplementary Figure 3S2**, **Supplementary Table S2**).

### PPI network construction of overlapping DEGs and hub genes identification

**Figure 5:**
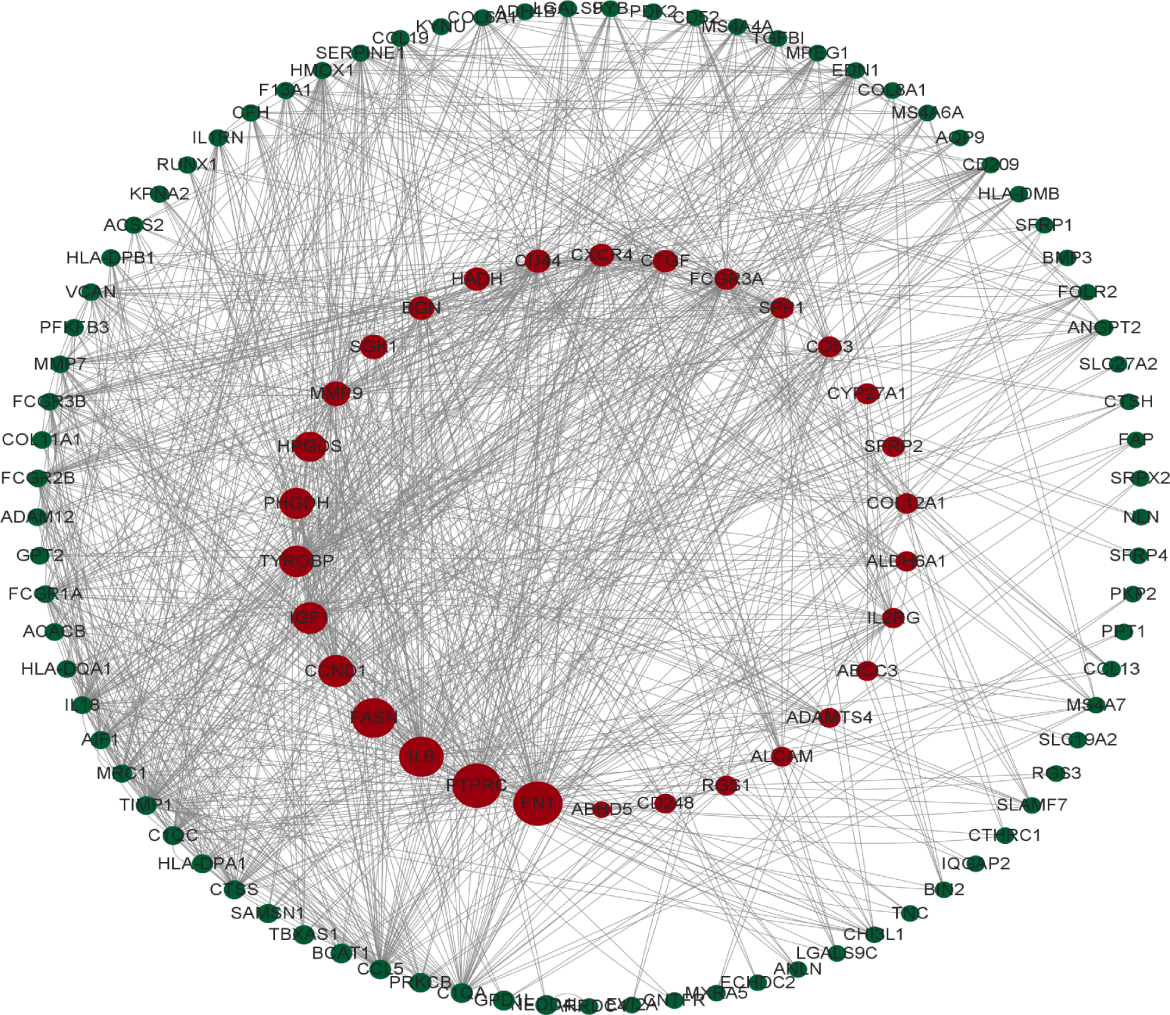
The protein interaction network obtained from the analysis of PPI with Cytoscape plugin cytoNCA, the circles represent the proteins and the lines connect the interacting proteins.

**Figure 6:**
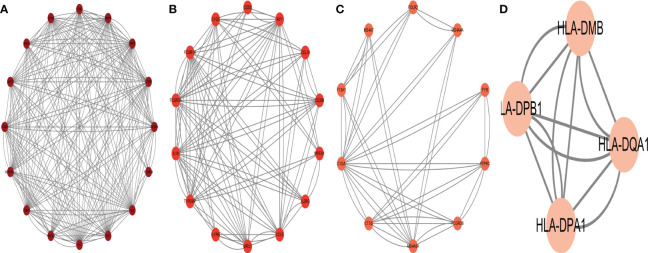
The protein interaction network obtained by analyzing PPI with Cytoscape plugin MCODE, **(A–D)** represent the four sub-modules obtained by MCODE plugin.

To identify the gene connections, the overlapping DEGs were imported into the STRING database (**Supplementary Figure 5S1**). Data findings were acquired, and Cytoscape software was used to perform a visual analysis. The Cytoscape plugin cytoNCA was used to examine the PPI network and find hub genes. (**Figure 5**). We examined the top 30 genes that might be crucial genes using the Median center algorithm: FN1, PTPRC, IL6, FASN, CCND1, IGF1, Tyrosine protein tyrosine kinase binding protein (TYROBP), PHGDH, HPGDS, MMP9, SGK1, BGN, HADH, CD44, CXCR4, CTGF, FCGR3A, SPP1, CD53, CYP27A1, SFRP2, COL12A1, ALDH6A1, IL2RG, ABCC3, ADAMTS4, ALCAM, RGS1, CD248, NEDD4L. In the meantime, we used the Cytoscape plugin MCODE to analyze the PPI network in order to find relevant gene cluster modules and calculate cluster scores (filter criteria: degree cut-off = 2, node score cut-off = 0.2, k-core = 2, max depth = 100). Five modules were obtained and four of them had module scores of at least four (**Figure 6**). We eliminated the top 30 candidates for hub genes: CXCR4, CTGF, FN1, IL6, CCND1, MMP9, SPP1, CD44, IGF1, CTGF, MMP7, EDN1, BGN, FCGR3B, TIMP1, SERPINE1, CD53, AIF1, CCL19, CD209, MPEG1, IL2RG, CCL5, MRC1, IL1RN, TYROBP, IL18, FCGR3A, FCGR1A, CC1QC.14 hub genes were produced when we combined the hub genes from cytoNCA and MCODE: TYROBP, CD44, BGN, FCGR3A, CD53, CXCR4, FN1, SPP1, IGF1, CCND1, MMP9, IL2RG, IL6, CTGF.

### Target transcription factors prediction

**Figure 7:**
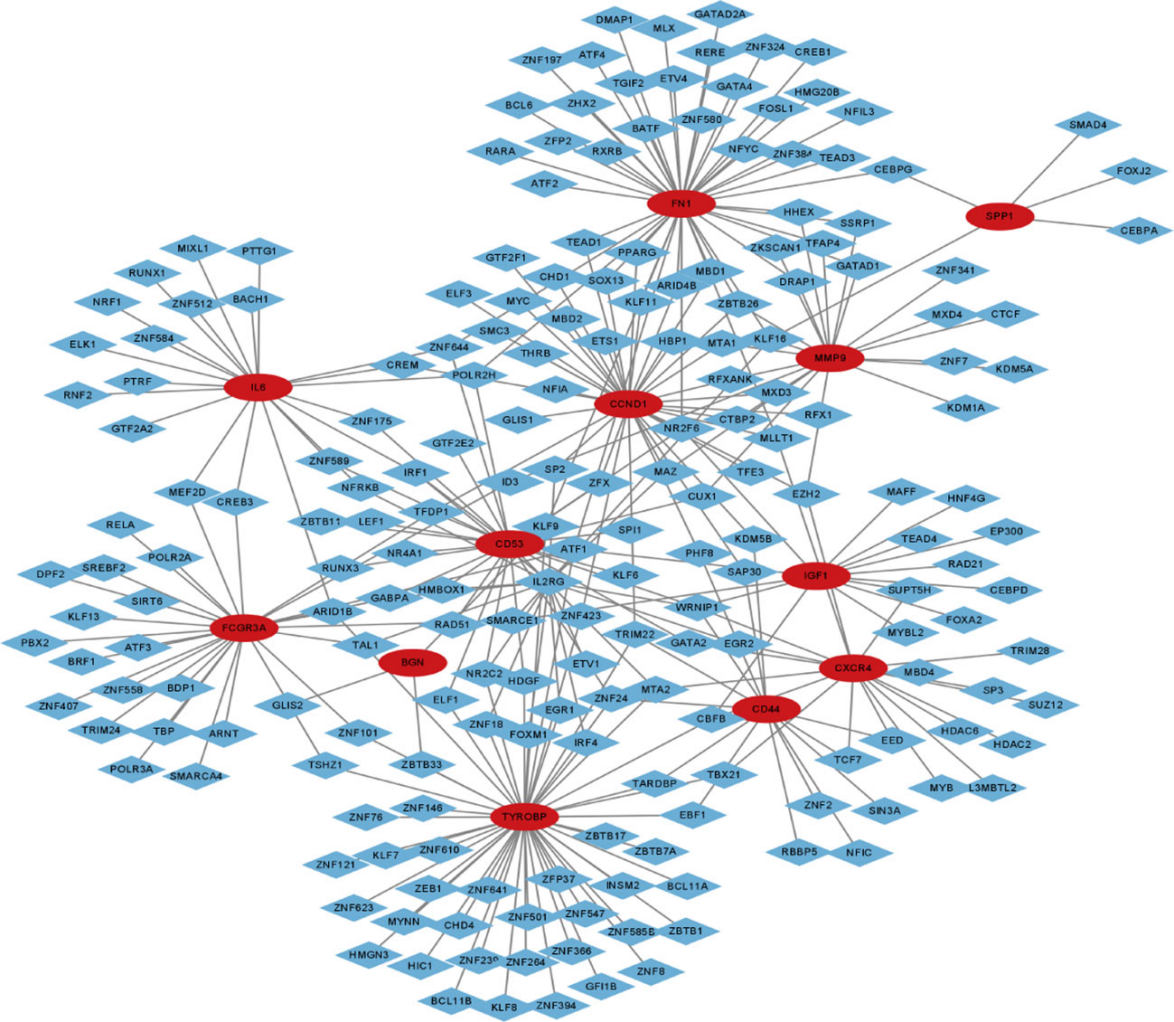
Transcription factor-gene network of 12 hub genes, red circles represent hub genes and blue diamonds represent transcription factors.

To forecast the hub genes’ target transcription factors, we used the Network Analyst database. Cytoscape software was used to import the obtained data results for visual analysis. In **Figure 7** and **Table 2**, we predicted the transcription factors of hub genes, where the transcription factor WRNIP1 interacts with 5 core genes, including CD44, CD53, IL2RG, IGF1, and CXCR4. Transcription factors ATF1, CBFB and NR2F6 interacted with 4 core genes, including transcription factor ATF1 interacting with FCGR3A, TYROBP, IGF1 and FN1. The transcription factor CBFB interacts with CD44, TYROBP, IGF1 and CXCR4. The transcription factor NR2F6 interacts with IGF1, CCND1, CD53 and FN1.The transcription factor ZNF24 interacts with CD44, TYROBP and CD53. The transcription factor NR2C2 interacts with IL2RG, TYROBP and CD53. The transcription factor SMARCE1 interacts with IL2RG, IGF1 and FCGR3A. The transcription factor GABPA interacts with FCGR3A, IGF1 and CD53. The transcription factor ARID1B interacts with FCGR3A, TYROBP and IL6. Transcription factor TFDP1 interacts with FCGR3A, CCND1 and CD53. The transcription factor MAZ interacts with IL2RG, CCND1 and MMP9. The transcription factor MLLT1 interacts with FN1, CCND1 and CXCR4. The transcription factors POLR2H and ZNF644 both interact with IL6, FN1 and CD53. The transcription factor ZBTB26 interacts with MMP9, FN1 and CCND1. The transcription factor KLF16 interacts with IL2RG, FN1 and SPP1. Transcription factor KLF9 interacts with BGN, TYROBP and FN1. The transcription factor EZH2 interacts with CXCR4, CCND1 and MMP9. Transcription factor TRIM22 interacts with CD44, CCND1 and TYROBP. The transcription factor MTA2 interacts with CD53, TYROBP and CXCR4.

**Table 2:** The transcription factors of identified hub genes.

Identified genes	Transcription factors
CCND1	TFE3, MTA1, MBD2, SOX13, HBP1, MXD3, ELF3, KDM5B, NFIA, CREM, MBD1, MYC, ZNF423, ARID4B, TFDP1, KLF11, ETS1, MAZ, SP2, EZH2, GTF2F1, RFXANK, THRB, CTBP2, GLIS1, ZFX, MLLT1, CHD1, NR2F6, SAP30, SMC3, RFX1, TRIM22, TEAD1, PPARG, ZBTB26, PHF8
CXCR4	EZH2, MLLT1, TCF7, GATA2, L3MBTL2, SUZ12, MYB, WRNIP1, TBX21, MTA2, SUPT5H, HDAC2, CBFB, TRIM28, HDAC6, SP3, EGR2, EED
BNG	ZBTB33, KLF9, GLIS2
FN1	KLF9, SOX13, MBD1, ARID4B, KLF11, MLLT1, NR2F6, TEAD1, PPARG, ZBTB26, BCL6, DRAP1, NFIL3, TEAD3, RXRB, NFYC, ZHX2, ZNF197, MLX, DMAP1, ZNF324, ETV4, CEBPG, FOSL1, TGIF2, HHEX, GATAD2A, TFAP4, GATA4, ZNF580, GATAD1, ATF1, HMG20B, POLR2H, ZNF384, RARA, RERE, BATF, SSRP1, KLF16, ZNF644, ATF2, ZFP2, ATF4, CREB1
IGF1	NR2F6, WRNIP1, SUPT5H, CBFB, ATF1, MAFF, FOXA2, RAD21, EP300, MYBL2, TEAD4, SMARCE1, CEBPD, MBD4, HNF4G
IL6	CREM, POLR2H, ZNF644, ARID1B, IRF1, MIXL1, CREB3, NRF1, GTF2A2, PTTG1, RNF2, RUNX1, BACH1, NFRKB, ELK1, PTRF, MEF2D, ZNF512, ZNF584, ZNF175, ZNF589
MMP9	MTA1, MXD3, MAZ, EZH2, RFXANK, CTBP2, ZBTB26, DRAP1, HHEX, TFAP4, GATAD1, SSRP1, KLF16, CTCF, MXD4, CUX1, KDM5A, ID3, ZNF7, ZKSCAN1, KDM1A, ZNF341
CD44	KDM5B, SAP30, TRIM22, PHF8, EED, EBF1, RBBP5, TCF7, NFIC, CBFB, ZNF24, SIN3A, ZNF2, TARDBP, WRNIP1
CD53	TFDP1, ZFX, NR2F6, ZNF24, WRNIP1, LEF1, ZBTB11, ELF1, HMBOX1, GATA2, KLF6, CUX1, SPI1, HDGF, GTF2E2, RAD51, ZNF644, MTA2, POLR2H, NR2C2, IRF1, ZNF175, GABPA, NR4A1, ZNF589, RUNX3
FCGR3A	TFDP1, GABPA, BDP1, TAL1, ZNF558, POLR3A, SMARCE1, ATF1, POLR2A, CREB3, BRF1, SMARCA4, SIRT6, SREBF2, DPF2, KLF13, TBP, TSHZ1, ATF3, ARID1B, ZNF407, ID3, ARNT, ZNF101, TRIM24, PBX2, MEF2D, RELA
SPP1	CEBPG, KLF16, FOXJ2, CEBPA, SMAD4
TYROBP	ZBTB33, KLF9, ZNF423, TRIM22, TBX21, MTA2, CBFB, ATF1, ARID1B, IRF4, FOXM1, EGR1, ZNF24, ZNF101, KLF7, HDGF, CHD4, ZNF641, ZNF264, ZNF121, ZNF366, ZBTB1, ZNF239, NR2C2, ZNF501, ZNF547, TSHZ1, GFI1B, ZNF585B, MYNN, ZNF76, BCL11A, ZNF610, ZNF394, ZFP37, HIC1, BCL11B, ZBTB7A, EBF1, ZBTB17, ELF1, ZNF18, HMGN3, ZNF623, ZNF146, TARDBP, INSM2, ZEB1, ZNF8, KLF8

### Target miRNAs prediction

**Figure 8:**
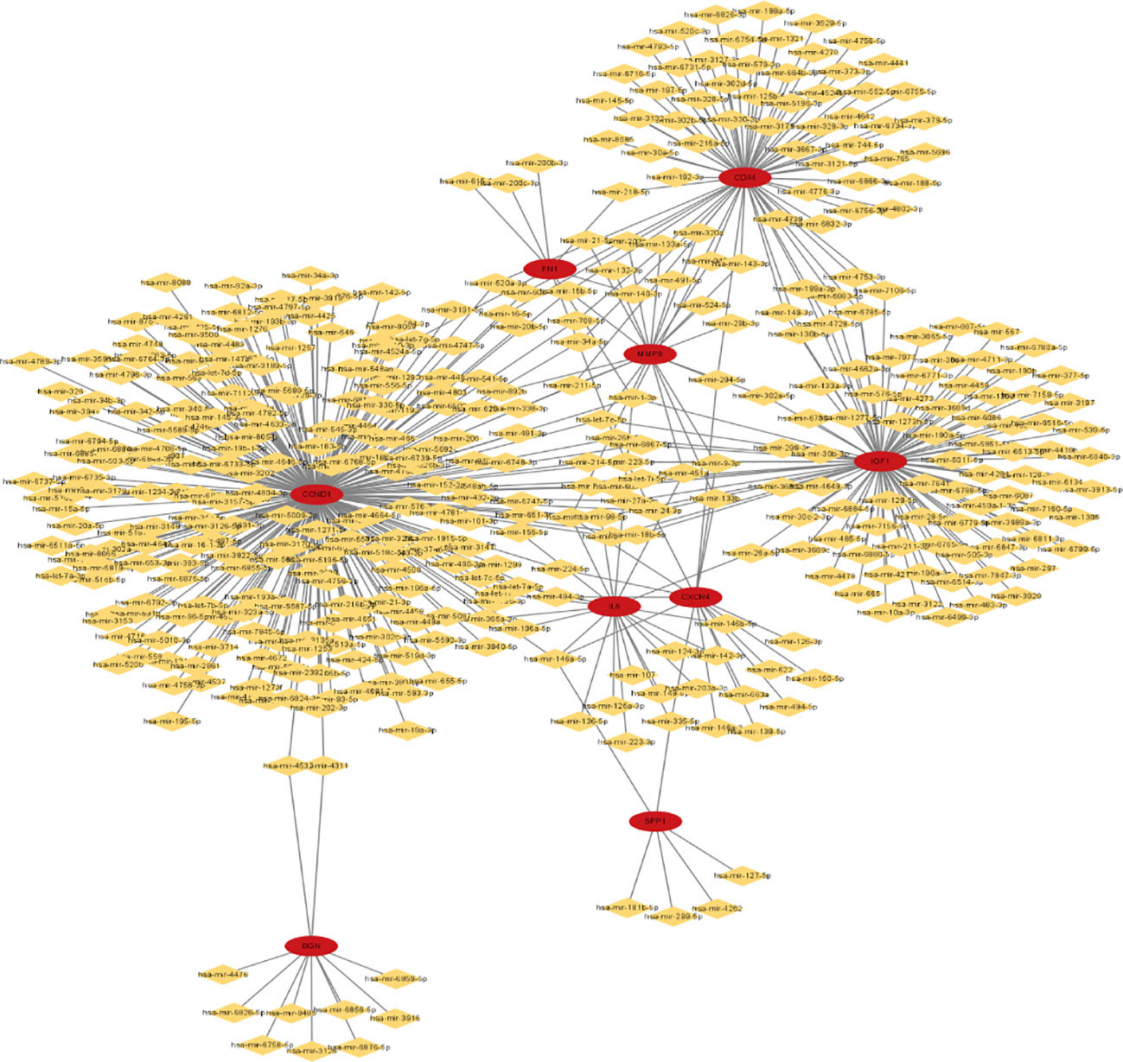
miRNA-gene network of 9 hub genes, red circles represent hub genes, yellow diamonds represent miRNAs.

To forecast the hub genes’ target transcription factors, we used the Network Analyst database. Cytoscape software was used to import the obtained data results for visual analysis. In **Figure 8** and **Table 3**, we predicted the transcription factors of hub genes, where the transcription factor WRNIP1 interacts with 5 core genes, including CD44, CD53, IL2RG, IGF1, and CXCR4.hsa-mir-26b-5p interacted with 4 central genes, including FN1, CCND1, CXCR4 and IGF1. hsa-mir-164a-5p interacted with 4 central genes, including IL6, SPP1, CCND1 and CXCR4. hsa-mir-9-5p interacts with 4 central genes, including IL6, MMP9, CCND1 and CXCR4. In addition, hsa-mir-98-5p interacts with IL6, CCND1 and IGF1. hsa-mir-204-5p interacts with MMP9, CD44 and CXCR4. hsa-mir-204-5p interacts with MMP9, CD44 and CXCR4. interacts with MMP9, CD44 and CXCR4. hsa-mir-211-5p interacts with MMP9, CD44 and CXCR4. hsa-mir-16b-5p interacts with MMP9, CD44 and CCND1. hsa-let-7e-5p interacts with MMP9, IGF1 and CCND1. hsa-mir-98-5p interacts with MMP9, CD44 and CCND1. hsa-mir-98-5p interacts with MMP9, CD44 and CCND1.

**Table 3:** The microRNA of identified hub genes.

Identified genes	microRNA
CCND1	hsa-let-7a-5p, hsa-let-7b-5p, hsa-let-7c-5p, hsa-let-7d-5p, hsa-let-7e-5p, hsa-let-7f-5p, hsa-mir-15a-5p, hsa-mir-16-5p, hsa-mir-17-5p, hsa-mir-18a-5p, hsa-mir-19a-3p, hsa-mir-20a-5p, hsa-mir-24-3p, hsa-mir-26b-5p, hsa-mir-27a-3p, hsa-mir-29a-3p, hsa-mir-92a-3p, hsa-mir-93-5p, hsa-mir-95-3p, hsa-mir-96-5p, hsa-mir-98-5p , hsa-mir-101-3p, hsa-mir-106a-5p, hsa-mir-196a-5p, hsa-mir-147a, hsa-mir-34a-5p, hsa-mir-183-5p, hsa-mir-211-5p, hsa-mir-224-5p, hsa-let-7g-5p, hsa-let-7i-5p, hsa-mir-1-3p, hsa-mir-15b-5p, hsa-mir-138-5p, hsa-mir-142-5p, hsa-mir-152-3p, hsa-mir-9-5p, hsa-mir-146a-5p, hsa-mir-193a-3p, hsa-mir-195-5p, hsa-mir-206, hsa-mir-155-5p, hsa-mir-106b-5p, hsa-mir-302a-3p, hsa-mir-34c-5p, hsa-mir-365a-3p, hsa-mir-302c-3p, hsa-mir-369-3p, hsa-mir-374a-5p, hsa-mir-380-5p, hsa-mir-383-5p, hsa-mir-326, hsa-mir-338-3p, hsa-mir-424-5p, hsa-mir-18b-5p, hsa-mir-20b-5p, hsa-mir-448, hsa-mir-449a, hsa-mir-490-3p, hsa-mir-202-3p, hsa-mir-432-3p, hsa-mir-494-3p, hsa-mir-193b-3p, hsa-mir-497-5p, hsa-mir-520a-3p, hsa-mir-526b-3p, hsa-mir-520b, hsa-mir-518c-5p, hsa-mir-519d-3p, hsa-mir-516b-5p, hsa-mir-503-5p, hsa-mir-513a-5p, hsa-mir-545-3p, hsa-mir-556-5p, hsa-mir-558, hsa-mir-563, hsa-mir-593-5p, hsa-mir-603, hsa-mir-608, hsa-mir-625-5p, hsa-mir-646, hsa-mir-425-5p, hsa-mir-668-3p, hsa-let-7a-3p, hsa-mir-16-1-3p, hsa-mir-19b-1-5p, hsa-mir-21-3p, hsa-mir-34a-3p, hsa-mir-214-5p, hsa-mir-223-5p, hsa-mir-145-3p, hsa-mir-34b-3p, hsa-mir-340-5p, hsa-mir-330-5p, hsa-mir-342-5p, hsa-mir-323a-5p, hsa-mir-491-3p, hsa-mir-574-5p, hsa-mir-576-3p, hsa-mir-593-3p, hsa-mir-298, hsa-mir-891b, hsa-mir-892b, hsa-mir-541-5p, hsa-mir-875-3p, hsa-mir-876-3p, hsa-mir-708-5p, hsa-mir-374b-5p, hsa-mir-944, hsa-mir-1234-3p, hsa-mir-1271-5p, hsa-mir-548k, hsa-mir-1293, hsa-mir-1299, hsa-mir-1243, hsa-mir-1253, hsa-mir-1257, hsa-mir-1276, hsa-mir-1915-5p, hsa-mir-2861, hsa-mir-3126-5p, hsa-mir-3135a, hsa-mir-466, hsa-mir-3147, hsa-mir-3148, hsa-mir-3153, hsa-mir-3157-5p, hsa-mir-3170, hsa-mir-1193, hsa-mir-323b-3p, hsa-mir-3179, hsa-mir-3202, hsa-mir-4311, hsa-mir-4261, hsa-mir-3609, hsa-mir-3648, hsa-mir-3663-5p, hsa-mir-3714, hsa-mir-3907, hsa-mir-3919, hsa-mir-676-5p, hsa-mir-3941, hsa-mir-4425, hsa-mir-548ah-5p, hsa-mir-4458, hsa-mir-4464, hsa-mir-4483, hsa-mir-4500, hsa-mir-2392, hsa-mir-4524a-5p, hsa-mir-4533, hsa-mir-548an, hsa-mir-4537, hsa-mir-3189-5p, hsa-mir-3922-5p, hsa-mir-3940-5p, hsa-mir-3973, hsa-mir-4633-3p, hsa-mir-4643, hsa-mir-4646-5p, hsa-mir-4651, hsa-mir-4664-5p, hsa-mir-4666a-5p, hsa-mir-4666a-3p, hsa-mir-4672, hsa-mir-4691-5p, hsa-mir-4691-3p, hsa-mir-4694-3p, hsa-mir-4708-5p, hsa-mir-4709-3p, hsa-mir-4715-5p, hsa-mir-4716-3p, hsa-mir-4720-3p, hsa-mir-4731-5p, hsa-mir-4735-3p, hsa-mir-3591-3p, hsa-mir-4746-3p, hsa-mir-4747-5p, hsa-mir-4748, hsa-mir-4756-3p, hsa-mir-4758-3p, hsa-mir-4781-3p, hsa-mir-4782-5p, hsa-mir-4789-3p, hsa-mir-4796-3p, hsa-mir-4797-5p, hsa-mir-4803, hsa-mir-4804-3p, hsa-mir-1273f, hsa-mir-5009-3p, hsa-mir-5010-3p, hsa-mir-5087, hsa-mir-5196-5p, hsa-mir-5197-3p, hsa-mir-4524b-5p, hsa-mir-5572, hsa-mir-5587-5p, hsa-mir-5589-5p, hsa-mir-5590-3p, hsa-mir-548av-5p, hsa-mir-5692c, hsa-mir-5692b, hsa-mir-5706, hsa-mir-204-3p, hsa-mir-3191-5p, hsa-mir-758-5p, hsa-mir-6077, hsa-mir-6500-5p, hsa-mir-6500-3p, hsa-mir-6511a-5p, hsa-mir-651-3p, hsa-mir-653-3p, hsa-mir-655-5p, hsa-mir-216b-3p, hsa-mir-1910-3p, hsa-mir-5699-5p, hsa-mir-6733-5p, hsa-mir-6735-3p, hsa-mir-6737-5p, hsa-mir-6739-5p, hsa-mir-6747-5p, hsa-mir-6748-3p, hsa-mir-6764-3p, hsa-mir-6768-5p, hsa-mir-6792-3p, hsa-mir-6794-5p, hsa-mir-6812-5p, hsa-mir-6818-5p, hsa-mir-6819-5p, hsa-mir-6824-3p, hsa-mir-6838-5p, hsa-mir-6843-3p, hsa-mir-6848-3p, hsa-mir-6855-5p, hsa-mir-6867-5p, hsa-mir-6873-5p, hsa-mir-6874-5p, hsa-mir-6875-5p, hsa-mir-6884-3p, hsa-mir-6885-3p, hsa-mir-7107-5p, hsa-mir-7112-3p, hsa-mir-7706, hsa-mir-7845-5p, hsa-mir-8054, hsa-mir-8066, hsa-mir-8068, hsa-mir-8069, hsa-mir-8080, hsa-mir-1199-3p, hsa-mir-9500
IL6	hsa-let-7a-5p, hsa-let-7c-5p, hsa-let-7f-5p, hsa-mir-26a-5p, hsa-mir-98-5p, hsa-mir-106a-5p, hsa-mir-107, hsa-mir-203a-3p, hsa-mir-223-3p, hsa-mir-1-3p, hsa-mir-124-3p, hsa-mir-142-3p, hsa-mir-9-5p , hsa-mir-136-5p, hsa-mir-146a-5p, hsa-mir-149-5p, hsa-mir-155-5p, hsa-mir-365a-3p, hsa-mir-335-5p, hsa-mir-451a, hsa-mir-146b-5p, hsa-mir-125a-3p
IGF1	hsa-let-7e-5p, hsa-mir-24-3p, hsa-mir-26a-5p, hsa-mir-26b-5p, hsa-mir-27a-3p, hsa-mir-28-5p, hsa-mir-29a-3p, hsa-mir-98-5p, hsa-mir-199a-3p, hsa-mir-129-5p, hsa-let-7i-5p, hsa-mir-1-3p, hsa-mir-128-3p, hsa-mir-133a-3p, hsa-mir-190a-5p, hsa-mir-302a-5p , hsa-mir-299-3p, hsa-mir-130b-3p, hsa-mir-18b-5p, hsa-mir-483-3p, hsa-mir-485-5p, hsa-mir-505-3p, hsa-mir-539-5p, hsa-mir-567, hsa-mir-576-5p, hsa-mir-603, hsa-mir-421, hsa-mir-297, hsa-mir-30c-2-3p, hsa-mir-10a-3p, hsa-mir-214-5p, hsa-mir-223-5p, hsa-mir-30b-3p, hsa-mir-149-3p, hsa-mir-30c-1-3p, hsa-mir-130b-5p , hsa-mir-377-5p, hsa-mir-455-3p, hsa-mir-574-5p, hsa-mir-190b, hsa-mir-665, hsa-mir-1305, hsa-mir-3122, hsa-mir-3065-5p, hsa-mir-3197, hsa-mir-4273, hsa-mir-4284, hsa-mir-3689a-3p, hsa-mir-3689b-3p, hsa-mir-3913-5p, hsa-mir-3929, hsa-mir-4459, hsa-mir-4478, hsa-mir-3689c, hsa-mir-3689d, hsa-mir-4419b , hsa-mir-4649-3p, hsa-mir-4662a-3p, hsa-mir-4711-3p, hsa-mir-4728-5p, hsa-mir-4753-3p, hsa-mir-5011-5p, hsa-mir-211-3p, hsa-mir-450a-1-3p, hsa-mir-1277-5p, hsa-mir-6086, hsa-mir-6087, hsa-mir-6134, hsa-mir-6499-3p, hsa-mir-6513-5p, hsa-mir-6514-3p, hsa-mir-190a-3p, hsa-mir-887-5p , hsa-mir-6739-3p, hsa-mir-6765-5p, hsa-mir-6771-3p, hsa-mir-6779-5p, hsa-mir-6780a-5p, hsa-mir-6785-5p, hsa-mir-6788-5p, hsa-mir-6799-5p, hsa-mir-6811-3p, hsa-mir-6840-3p, hsa-mir-6847-3p, hsa-mir-6851-5p, hsa-mir-6867-5p, hsa-mir-6880-5p, hsa-mir-6883-5p, hsa-mir-6884-5p, hsa-mir-7106-5p, hsa-mir-7156-5p, hsa-mir-7159-5p, hsa-mir-7160-5p, hsa-mir-7641, hsa-mir-1273h-5p, hsa-mir-6516-5p, hsa-mir-7847-3p, hsa-mir-7977
MMP9	hsa-let-7e-5p, hsa-mir-21-5p, hsa-mir-29b-3p, hsa-mir-204-5p, hsa-mir-211-5p, hsa-mir-15b-5p, hsa-mir-132-3p, hsa-mir-143-3p, hsa-mir-9-5p, hsa-mir-9-3p, hsa-mir-320a, hsa-mir-302a-5p, hsa-mir-338-3p, hsa-mir-133b, hsa-mir-451a, hsa-mir-491-5p, hsa-mir-524-5p, hsa-mir-892b, hsa-mir-133a-5p, hsa-mir-942-3p, hsa-mir-203a-5p
CD44	hsa-mir-16-5p, hsa-mir-30a-5p, hsa-mir-199a-5p, hsa-mir-199a-3p, hsa-mir-34a-5p, hsa-mir-204-5p, hsa-mir-211-5p, hsa-mir-216a-5p, hsa-mir-218-5p, hsa-mir-1-3p, hsa-mir-15b-5p, hsa-mir-125b-5p, hsa-mir-143-3p, hsa-mir-145-5p, hsa-mir-188-5p, hsa-mir-320a, hsa-mir-302b-5p, hsa-mir-373-3p, hsa-mir-379-5p, hsa-mir-330-3p, hsa-mir-328-3p, hsa-mir-520a-3p, hsa-mir-520c-3p, hsa-mir-579-3p, hsa-mir-608, hsa-mir-765, hsa-mir-192-3p, hsa-mir-149-3p, hsa-mir-130b-5p, hsa-mir-302d-5p, hsa-mir-708-5p, hsa-mir-744-5p, hsa-mir-1321, hsa-mir-3132, hsa-mir-3175, hsa-mir-4270, hsa-mir-3667-3p, hsa-mir-4441, hsa-mir-3121-5p, hsa-mir-3127-3p, hsa-mir-4642, hsa-mir-3529-5p, hsa-mir-4728-5p, hsa-mir-4739, hsa-mir-4753-3p, hsa-mir-4756-5p, hsa-mir-4776-3p, hsa-mir-4793-5p, hsa-mir-4802-3p, hsa-mir-5196-3p, hsa-mir-4524b-3p, hsa-mir-664b-3p, hsa-mir-5696, hsa-mir-197-5p, hsa-mir-6716-5p, hsa-mir-328-5p, hsa-mir-552-5p, hsa-mir-942-3p, hsa-mir-6731-5p, hsa-mir-6734-3p, hsa-mir-6754-5p, hsa-mir-6755-5p, hsa-mir-6756-3p, hsa-mir-6785-5p, hsa-mir-6826-3p, hsa-mir-6832-3p, hsa-mir-6866-3p, hsa-mir-6883-5p, hsa-mir-7106-5p, hsa-mir-8085
FN1	hsa-mir-26b-5p, hsa-mir-218-5p, hsa-mir-200b-3p, hsa-let-7g-5p, hsa-mir-1-3p, hsa-mir-200c-3p, hsa-mir-615-3p, hsa-mir-140-3p
CXCR4	hsa-mir-26b-5p, hsa-mir-139-5p, hsa-mir-204-5p, hsa-mir-224-5p, hsa-mir-9-5p, hsa-mir-9-3p, hsa-mir-126-3p, hsa-mir-146a-5p, hsa-mir-150-5p, hsa-mir-335-5p, hsa-mir-133b, hsa-mir-494-3p, hsa-mir-622, hsa-mir-663a, hsa-mir-146a-3p, hsa-mir-494-5p
SPP1	hsa-mir-181b-5p, hsa-mir-146a-5p, hsa-mir-335-5p, hsa-mir-299-5p, hsa-mir-127-5p, hsa-mir-4262
BGN	hsa-mir-3125, hsa-mir-4311, hsa-mir-3916, hsa-mir-4476, hsa-mir-4533, hsa-mir-6758-5p, hsa-mir-6828-5p, hsa-mir-6856-5p, hsa-mir-6859-5p, hsa-mir-6876-5p, hsa-mir-8485

### Transcription factors and miRNA network construction of hub genes

**Figure 9:**
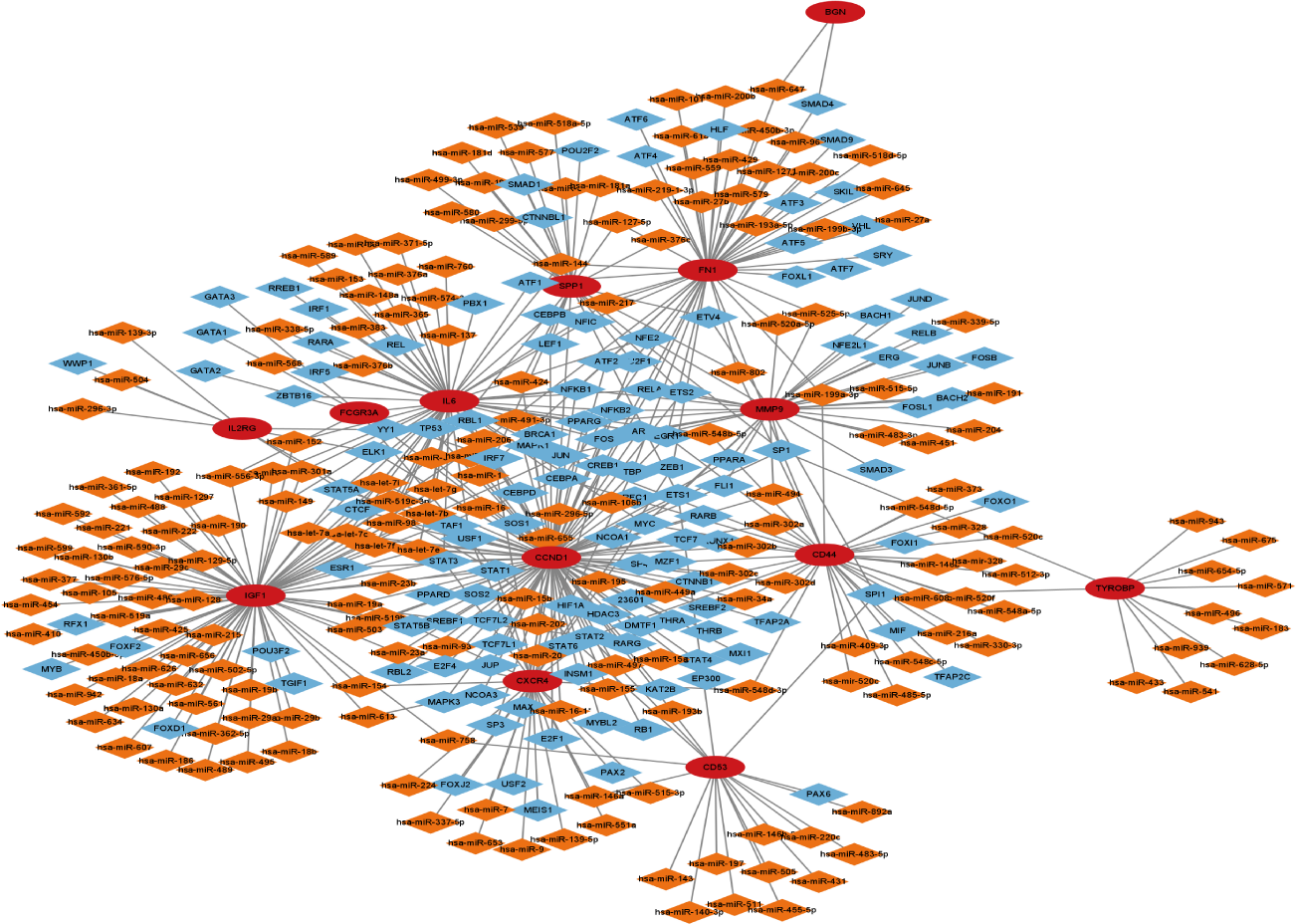
Transcription factor-miRNA-gene network of 13 hub genes, red circles represent hub genes, blue diamonds represent transcription factors, and orange diamonds represent miRNAs.

To build the network between target miRNA-transcription and factor-hub genes, we used the Network Analyst database. Cytoscape software was used to visualize and analyze the generated data results (**Figure 9**).

## Discussion

In practically all developing and developed nations, the prevalence of overweight and obesity has substantially increased. In developed nations, it has reached prevalence levels of 60–70% of the adult population and is particularly prevalent in cities and among women. Undoubtedly, this poses a significant threat to the weight of the global economy (28). While obesity-related endocrine disturbance is significantly linked to the onset of cancer, earlier research has found a connection between obesity and CC in males. It is still not entirely apparent how CC is related to obese females. Therefore, it is very important to investigate any potential biological processes that link female obesity to the development of CC. In this study dataset GSE44076, female CC patients had an average age of 69.63 ± 9.44 years. These female patients were diagnosed with CC when they were in the menopausal stage. Endocrine disturbance and the chronic inflammatory state brought on by weight changes in these patients may be directly related to the onset of CC at this period.

In this study, we downloaded data from two gene chips from the GEO website and performed a series of bioinformatic analyses based on obese women and women with CC. Between the two datasets, we were able to identify 146 DEGs. We discovered that these genes were primarily enriched in inflammatory and immune-related pathways by GO analysis and KEGG analysis. These pathways were tightly linked to CC development and endocrine dysregulation brought on by obesity. We also discovered 14 hub genes, including TYROBP, CD44, BGN, FCGR3A, CD53, CXCR4, FN1, SPP1, IGF1, CCND1, MMP9, IL2RG, IL6 and CTGF, which were crucial in the development of CC and obesity. Then, for these 14 hub genes, we created the miRNAs-gene-transcription factor regulatory networks.

Previous research has demonstrated that obesity and overweight also contribute to cancer. 20% of cancer deaths among women in the US are brought on by being overweight or obese (6). Obesity commonly causes metabolic problems, altered steroid hormone secretion and persistent subclinical inflammation, all of which are highly related with the development of cancer (29). Circulating estrogen levels in postmenopausal women are inversely correlated with fat mass. The primary source of the synthesis of circulating estrogen comes from androgens in adipose tissue. It has been demonstrated that greater estrogen levels are linked to obesity and overweight (30). Increased amounts of circulating estrogen alter the levels of cytokines, hormones and inflammatory markers, which may worsen the prognosis for cancer patients (31). Immune cells, including macrophages and lymphocytes, penetrate white adipose tissue in obese people. Obese fat pads resemble chronically injured tissue in this sense, and they can be a rich source of pro-inflammatory chemicals that may promote tumor growth (32). The adipose microenvironment in the obese population is comparable to the tumor microenvironment in many ways, including cellular composition, chronic low-grade inflammation and a high ratio of reactive oxygen species to antioxidants (33). These possible mechanisms are similar to the gene functions that we discovered to be frequently dysregulated in both datasets. Our findings also indicate that the overlapped gene functions in the two datasets are predominantly enriched in inflammatory and immune-related pathways. To summarize, obesity and cancer formation are inextricably linked because obesity’s endocrine disruption and chronic inflammatory state provide a potential material basis for tumor development. In the case of women, we must consider the unique circumstance of the patient’s menopausal status, since this condition is also linked to the development of obesity.

The NetworkAnalyst program was used to generate these hub gene-associated target miRNAs and transcription factors. The four most active miRNAs with the hub genes were miR-1-3p, miR-26b-5p, miR-164a-5p, and miR-9-5p. WRNIP1, ATF1, CBFB, and NR2F6 were the four transcription factors that interacted the most with the hub genes. MiRNAs serve important roles in cellular processes such as cellular differentiation, stress response, proliferation, and apoptosis. Abnormal miRNA expression is linked to carcinogenesis and cancer progression (34). MiRNAs are intriguing molecular biomarkers for cancer diagnosis, prognosis, and responsive therapeutic targets, including colon cancer (35). Transcription factors can be found in a variety of human organs and cells. To control transcription, recognize certain DNA sequences (36). We expected that the transcription factors WRNIP1, ATF1, CBFB, and NR2F6, as well as the microRNAs miR-1-3p, miR-26b-5p, miR-164a-5p, and miR-9-5p, would play essential roles in metabolic abnormalities of cells in obese people and the development of colon cancer.

However, our research is bound to have limitations. We were unable to identify a dataset on CC in the setting of female obesity to support our findings. In addition, we lack matching transcriptome sequencing data to back up our findings. Future research should look for equivalent validation datasets to validate the findings. As a result, subsequent pertinent experimental studies reveal possible diagnostic and therapeutic targets for female obesity-related CC patients.

In conclusion, our research discovered a number of critical genes. miRNAs - gene-transcription factor regulatory networks - play a role in the pathophysiology of female obesity and female CC, making them attractive diagnostic and therapeutic targets for CC in the setting of female obesity.

## Conclusion

Our findings show that females with obesity and females with CC have shared genes. Our findings suggest that obesity and CC can be genetically connected in female individuals. Between female adipose patients and female CC, we discovered 14 hub genes and built a transcription factor-miRNA-gene network comprising hub genes. WRNIP1, ATF1, CBFB, and NR2F6 transcription factors were found in our study. Furthermore, hsa-mir-1-3p, hsa-mir-26b-5p, hsa-mir-164a-5p, and hsa-mir-9-5p are implicated in the development of female obesity and female colon cancer via inflammatory and immune-related pathways. Our findings could help with future mechanistic research and medication target prediction.

## Data availability statement

The datasets presented in this study can be found in online repositories. The names of the repository/repositories and accession number(s) can be found in the article/**Supplementary Material**.

## Ethics statement

Ethical approval was not required for the study involving humans in accordance with the local legislation and institutional requirements. Written informed consent to participate in this study was not required from the participants or the participants’ legal guardians/next of kin in accordance with the national legislation and the institutional requirements. Written informed consent was not obtained from the individual(s) for the publication of any potentially identifiable images or data included in this article because Our raw data is obtained from a public database.

## Author contributions

X-LZ, H-YL and TY conceived and designed the study. YF, X-FZ, M-LL, RS and YG collected and analyzed the data. X-LZ wrote the original draft. H-YL and YT revised the manuscript. X-LZ, H-YL and YT reviewed and edited the manuscript. All authors contributed to the article and approved the submitted version. All authors contributed to the article and approved the submitted version.
