# Supplementary figures and images for: A possible genetic association between obesity and colon cancer in females
Source: Front Endocrinol (Lausanne). 2023 Aug 30;14:1189570. doi: 10.3389/fendo.2023.1189570 (PMC10497871; doi:10.3389/fendo.2023.1189570)

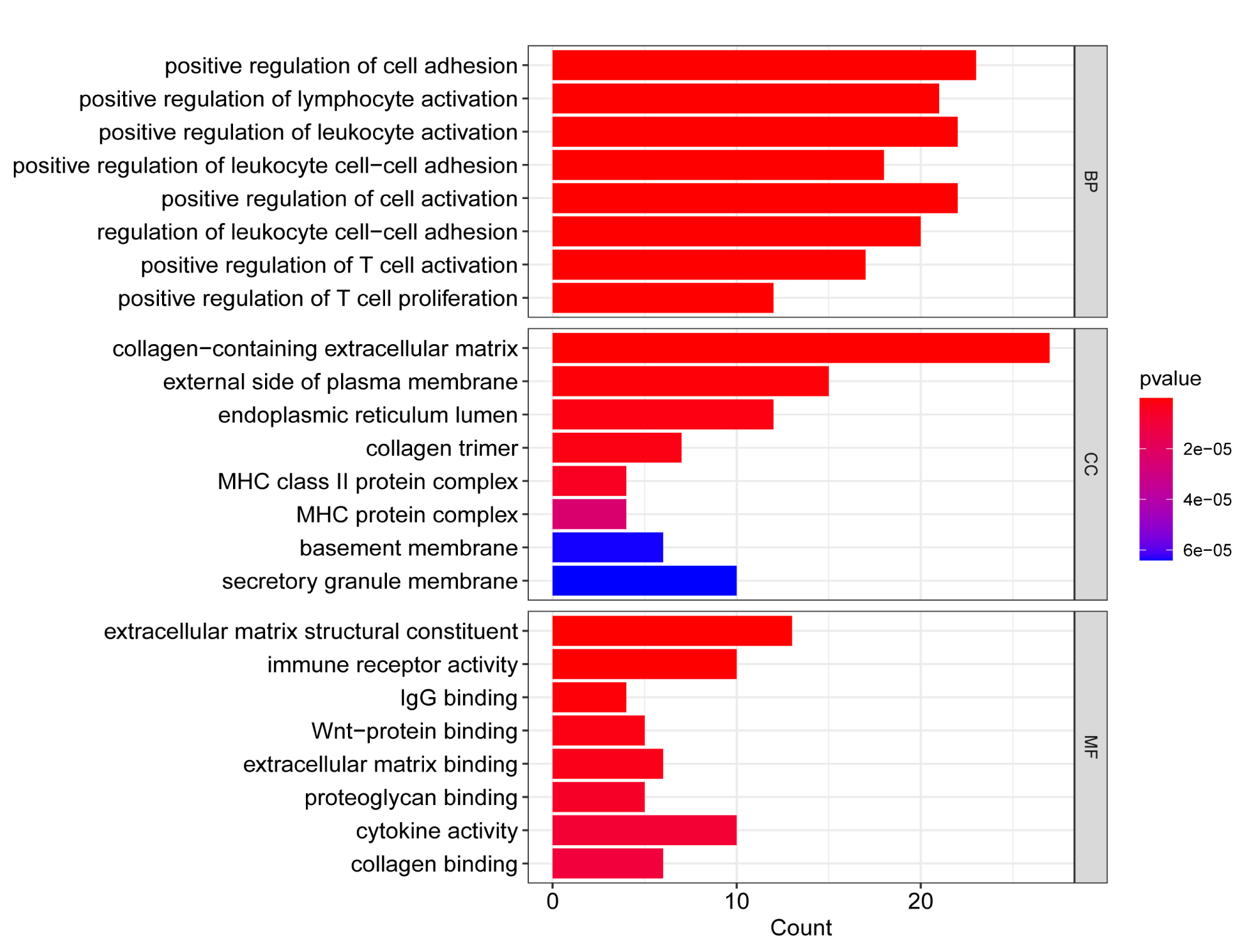

Supplement: Supplementary file 1 [file Image_1.tif]

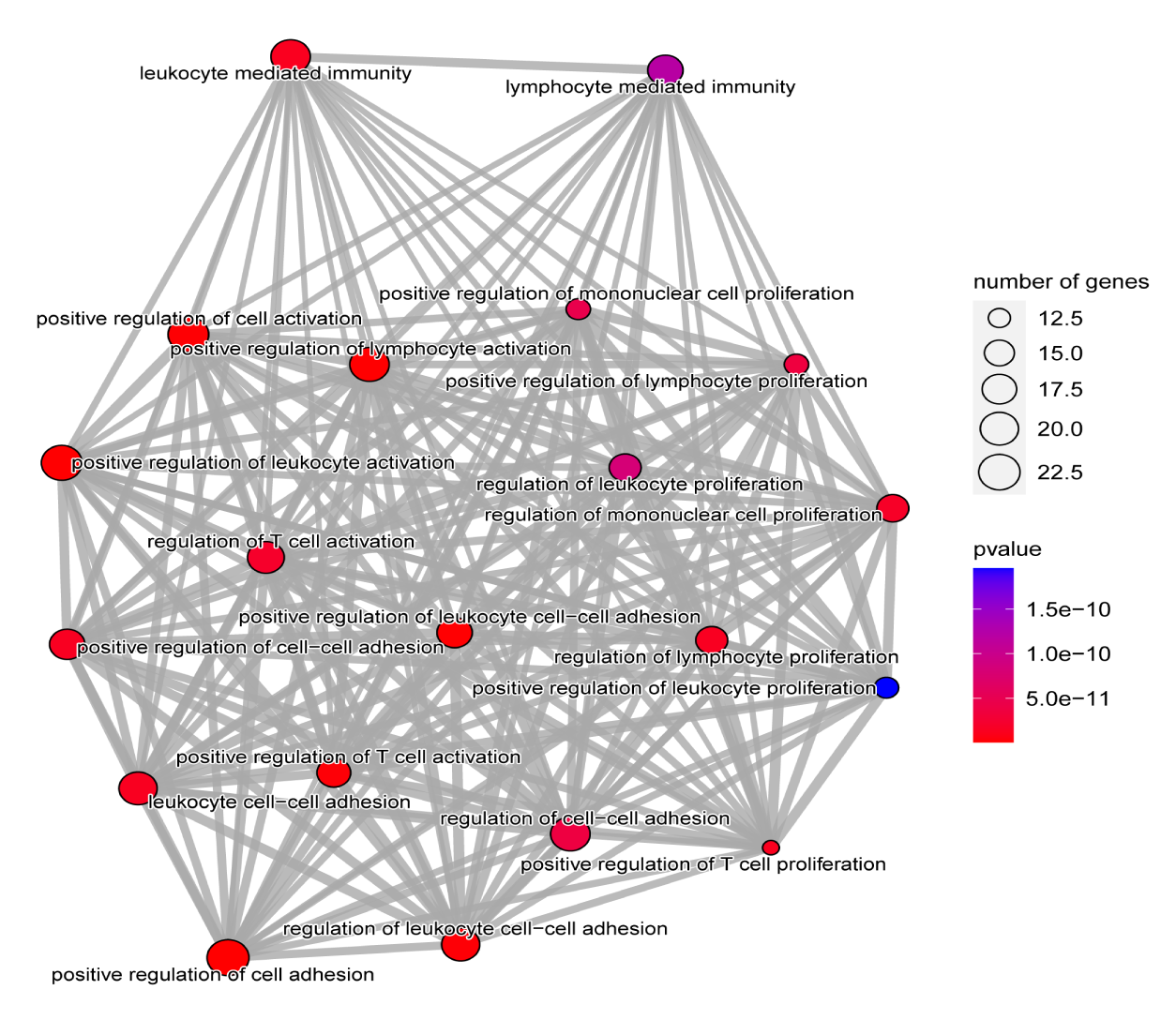

Supplement: Supplementary file 2 [file Image_2.tif]

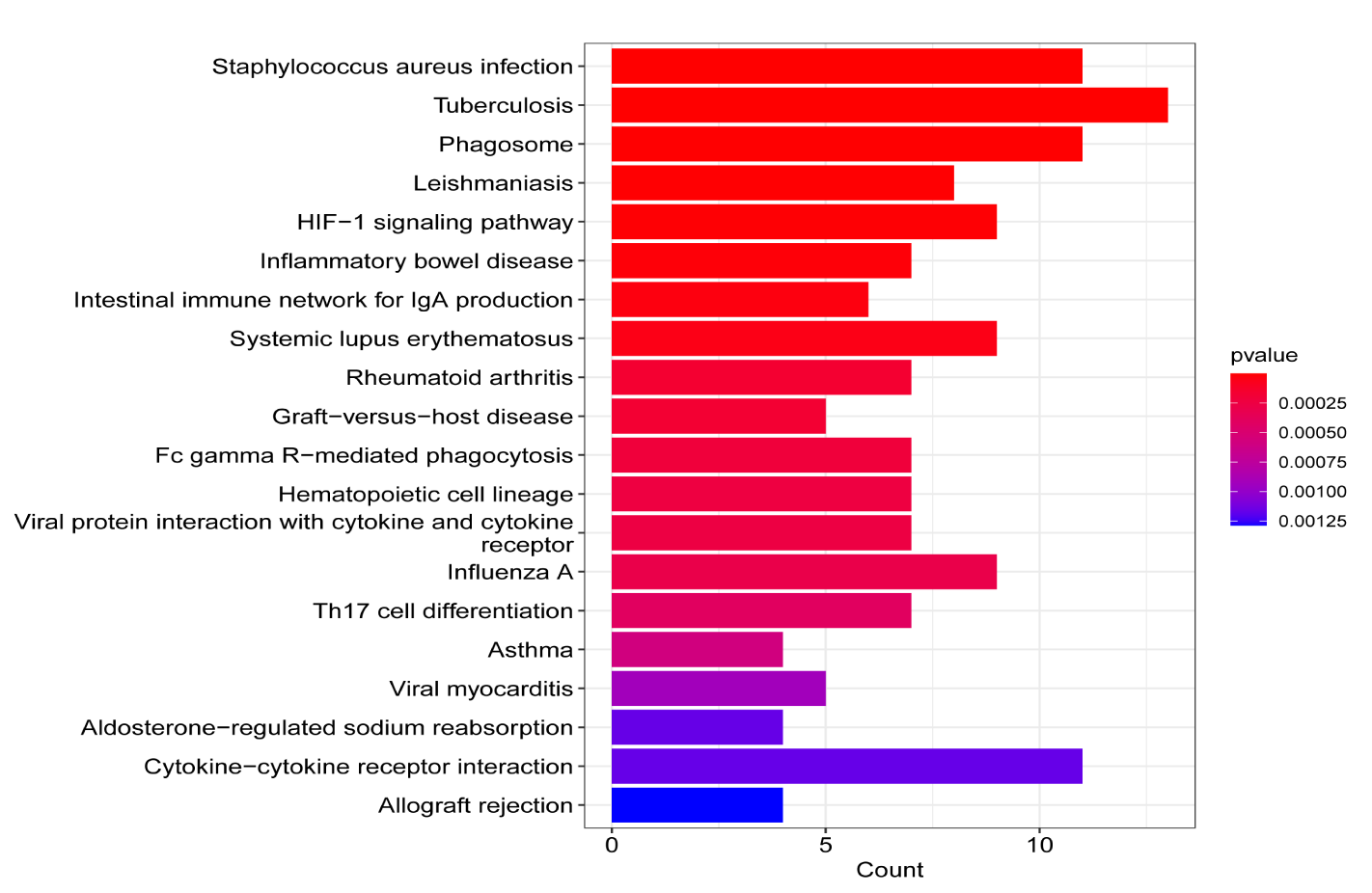

Supplement: Supplementary file 3 [file Image_3.tif]

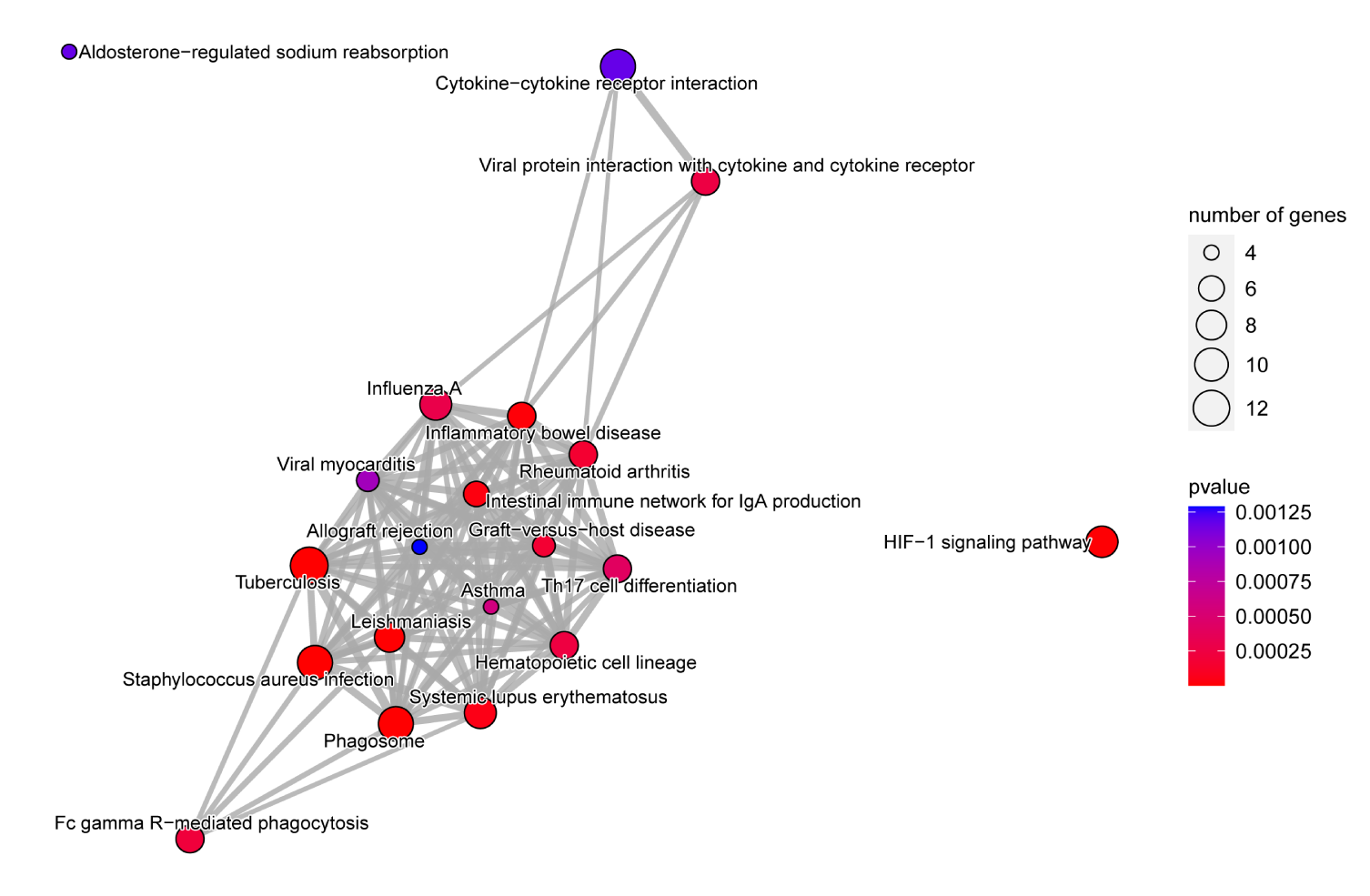

Supplement: Supplementary file 4 [file Image_4.tif]

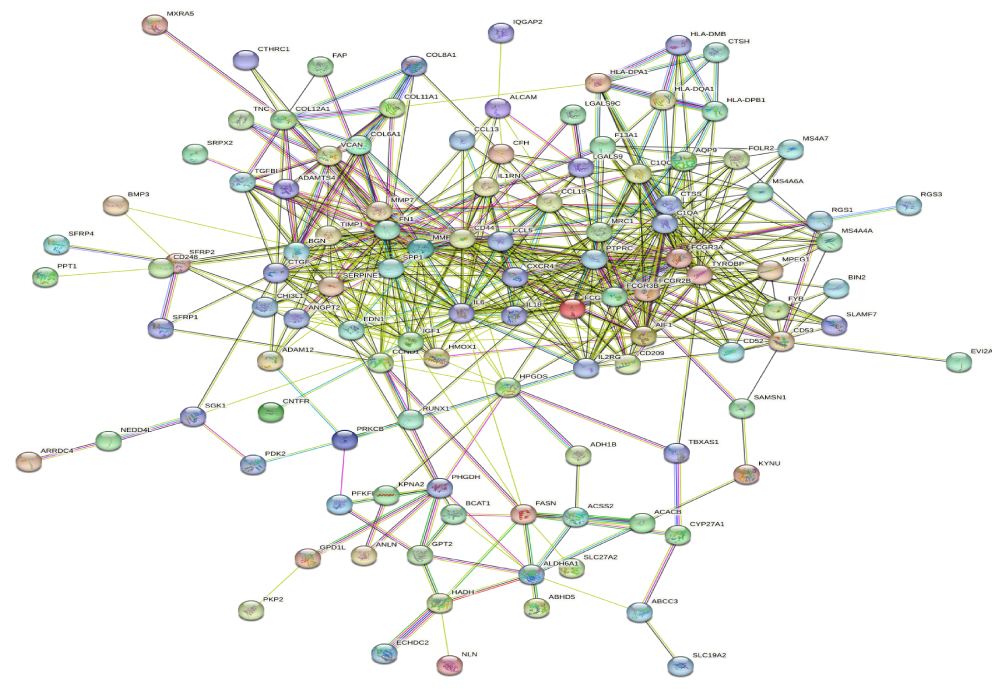

Supplement: Supplementary file 5 [file Image_5.tif]
